# Women and other risk factors for chronic kidney disease of unknown etiology in Thailand: National Health Examination V Survey

**DOI:** 10.1038/s41598-021-00694-9

**Published:** 2021-11-01

**Authors:** Wichai Aekplakorn, Suwat Chariyalertsak, Pattapong Kessomboon, Sawitri Assanangkornchai, Surasak Taneepanichskul, Nareemarn Neelapaichit, Anchalee Chittamma, Chagriya Kitiyakara

**Affiliations:** 1 Department of Community Medicine, Faculty of Medicine, Ramathibodi Hospital, Mahidol University, Bangkok, Thailand; 2 Faculty of Public Health, Chiang Mai University, Chiang Mai, Thailand; 3 Department of Community Medicine, Faculty of Medicine, Khon Kaen University, Khon Kaen, Thailand; 4 Epidemiology Unit, Faculty of Medicine, Prince of Songkla University, Songkhla, Thailand; 5 College of Public Health Sciences, Chulalongkorn University, Bangkok, Thailand; 6 Ramathibodi School of Nursing, Faculty of Medicine, Ramathibodi Hospital, Mahidol University, Bangkok, Thailand; 7 Department of Pathology, Faculty of Medicine, Ramathibodi Hospital, Mahidol University, Bangkok, Thailand; 8 Department of Medicine, Faculty of Medicine, Ramathibodi Hospital, Mahidol University, Bangkok, Thailand

**Keywords:** Nephrology, Chronic kidney disease, Epidemiology

## Abstract

There are limited data on chronic kidney disease of unknown etiology (CKDu) from Southeast Asia. Initially described in working age men, a common approach to detect CKDu that includes all adults has recently been proposed. We determined the prevalence, and risk factors for CKDu using data from a cross-sectional, nationally representative survey of the adult population of Thailand. We used a proxy for CKDu as age < 70 with impaired kidney function (eGFR < 60) in the absence of diabetes and hypertension (CKDu1) and heavy proteinuria (CKDu2). Prevalence estimates were probability-weighted for the Thai population. The associations between risk factors and CKDu or elderly subjects with eGFR < 60 without traditional causes were assessed by multivariable logistic regression. Of 17,329 subjects, the prevalence were: eGFR < 60, 5.3%; CKDu1 0.78%; CKDu2, 0.75%. CKDu differed by 4.3-folds between regions. Women, farmers/laborers, older age, gout, painkillers, rural area, and stones were independent risk factors for CKDu. Women, age, rural, gout, painkillers were significant risk factors for both CKDu and elderly subjects. These data collected using standardized methodology showed that the prevalence of CKDu in Thailand was low overall, although some regions had higher risk. Unlike other countries, Thai women had a two-fold higher risk of CKDu.

## Introduction

The prevalence of chronic kidney disease (CKD) in low and middle-income countries (LMIC) has surpassed that of developed countries^1,2^. A new form of severe CKD without common underlying factors, such as diabetes, hypertension, or glomerulonephritis named CKD of unknown etiology (CKDu), has emerged over the last 20 years^3,4^. This disorder could be fatal due to late recognition. CKDu is strongly associated with a hot climate, but whether the cause is heat stress, drugs, agrochemicals, water contaminants, or a combination of factors is not yet known. Most data has come from Central America and South Asia, where the disease has reached epidemic proportions predominantly affecting men in agricultural communities and laborers^3,4^. Currently, it is uncertain where CKDu might be highly prevalent, and whether the etiologies are the same in different parts of the world.

Kidney biopsies in CKDu show predominantly chronic tubulointerstitial disease^5^. In epidemiological studies, clinical criteria are used to detect CKDu. Ascertaining the prevalence for CKDu has been difficult because the criteria for CKDu had not been fully agreed upon. Recently, the International Society of Nephrology’s International Consortium of Collaborators on Chronic Kidney Disease of Unknown Etiology (i3C) formulated a common approach to detect CKDu^6^. The proxy definition for suspected CKDu in epidemiologic studies required a single measurement of eGFR < 60 using standardized creatinine, and the exclusion of diabetes or hypertension, or heavy proteinuria. The ISN proxy for suspected CKDu did not set an upper age limit whereas initial reports of CKDu mostly focused on working-age adults, and excluded subjects above age 70 years old^7^. Although these definitions given as examples by the ISN i3C and proposed by the Disadvantaged Populations eGFR Epidemiology (DEGREE) collaboration probably give us the best insight into the burden of CKDu, they have not yet been mandated by professional organizations^8^. Whether elderly subjects (age ≥ 70) fulfilling the CKDu proxy criteria share the same risk factors as CKDu (age < 70) has not been fully explored.

Most data on CKDu has come from communities with few nationally-representative studies. Moreover, there are limited data from Southeast Asia. Thailand is a Southeast Asian country with a population of over 65 million people. In 2011, the World Bank reclassified Thailand to a high-middle income country. In the past, Thailand had among the highest prevalence of CKD in the world^9,10^, and the numbers with kidney failure had increased 18 folds in a decade^11^. There are limited information on CKDu, which may be particularly high since many people are farmers or laborers. We hypothesized that there is likely to be important variations in CKDu prevalence in Thailand given a significant degree of socioeconomic disparity between urban and rural areas, and between different regions despite overall economic development. Moreover, the risk factors for CKDu in Thailand may differ from other parts of the world. For the purposes of this study, we limited the term ‘CKDu’ to subjects age < 70 since this reflects the initial concept of CKDu^7^. We plan (1) to assess the prevalence, regional distribution, and risk factors of CKD and CKDu in the adult general population of Thailand from the National Health Examination V (NHES V) survey and compare these with elderly subjects (age ≥ 70) with decreased kidney function without traditional causes (hypertension, diabetes (or heavy proteinuria)), and (2) to evaluate the awareness of CKDu.

## Material and methods

### Sampling methods

The NHES V was a cross-sectional survey of a representative non-institutionalized Thai population conducted in 2014^12^. In brief, the survey applied a four-stage sampling method as follows: (1) five provinces in each of the four regions of Thailand, and Bangkok, were selected; (2) two to three districts were randomly selected from each province; (3) 24 enumeration areas (EAs) randomly selected from each province, with 12 EAs in urban and 12 EAs in rural areas, for a total 540 EAs; (4) individuals of both sexes from each age group (15–29, 30–44, 45–59, 60–69, 70–79, and 80 years or more) were randomly selected from each EA. Of the planned 22,095 participants (aged ≥ 20 years), 18,118 adults participated in the survey (82% response rate).

### Data collection

The survey was approved by the Ethical Review Committee for Research in Human Subjects, Faculty of Medicine, Ramathibodi Hospital, Mahidol University and all methods were carried out in accordance with the Declaration of Helsinki. Each subject gave written informed consent. Data were collected in face-to-face interviews conducted by research nurses using a questionnaire at community centers. History of cardiovascular disease (myocardial infarction or stroke), urinary stones, gout, use of pain killers, and herbal medicines were noted. Subjects were classified as agricultural workers or laborers according to their response to the occupation question.

Participants were categorized by body mass index (kg/m^2^) according to World Health Organization criteria. Blood pressure was measured using an automatic blood pressure monitor (Omron model HEM-7117, Omron HealthCare Co., Ltd., Kyoto, Japan)^6^. Three serial blood pressure measurements were made after at least 5 min of rest. The average of the second and third reading was used. The research team was given an instruction manual and completed a training program on the study process.

### Definitions of covariates

Diabetes was defined as fasting plasma glucose (FPG) of 126 mg/dl or more and/or use of antihypoglycemic medications. Hypertension was defined as either systolic blood pressure 140 mm Hg or diastolic blood pressure of 90 mm Hg, or more and/or use of antihypertensive medications.

### Laboratory measurements

All blood and urine samples except serum creatinine were analyzed at the laboratory in each province. All laboratories completed a standardization and certification program. FPG was measured using an enzymatic hexokinase method. Urine protein was detected by urinalysis reagent strip (Bayer, Indiana, USA).

### Creatinine measurements and estimated glomerular filtration rate (eGFR) calculation

Serum creatinine (sCr) was measured in a central laboratory of Ramathibodi Hospital on the Vitros 350 analyzer (Ortho-Clinical Diagnostics, USA. Calibration was performed using two levels of IDMS-Standard Reference Material (SRM) 967 (CV%: low, 1.64%; and high, 0.41%) as published^13^.

**e**GFR (ml/min/1.73 m^2^) was calculated from standardized creatinine using Chronic Kidney Disease–Epidemiology Collaboration CKD-EPI equation for non-blacks^14^, since this is the preferred equation of the Nephrology Society of Thailand, and the ISN i3C workgroup to allow international comparisons^6^. Additional analyses were also performed with the Modification of diet in renal disease (MDRD) study equation^15^, and with the Thai GFR (T-GFR) equation^16^.

#### Staging and definitions for CKD

Subjects were divided into stages by eGFR (G1–5) and albuminuria (A1–3) according to the 2012 KDIGO guidelines^17^. Stage 3 was subdivided into stages 3a and 3b with a cutoff of eGFR = 45. Dipstick protein was used instead of albuminuria data as follows: A1, dipstick negative or trace; A2, 1 + (mild); A3, protein ≥ 2 + (moderate to severe proteinuria). The CKD diagnosis by KDIGO requires that abnormal kidney function be present for at least 3 months. Since repeated testing is impractical, we used proxy definitions based on one-time kidney function testing as recommended by the i3C workgroup^6^ as follows:

- *Proteinuria* A2 or A3 regardless of G stage.
- *No CKD* eGFR > 60 and no proteinuria.
- *CKD1-5* eGFR < 60 and/or proteinuria.

Decreased GFR *(eGFR* < *60)* as a proxy for CKD stage 3–5.

Decreased GFR without traditional causes were divided into:

1. Decreased GFR without diabetes or hypertension *(eGFR* < *60(DM-HT-))* were categorized as *CKDu1,* if age < 70, and as *eGFR* < *60 (DM-HT-)*_*Age*≥*70*_, if Age ≥ 70.
2. Decreased GFR without diabetes, hypertension, or severe proteinuria (no proteinuria A3) *(eGFR* < *60 (DM-HT-Prot* ≤ *1* +*))* were categorized as *CKDu2,* if age < 70, and as *eGFR* < *60 (DM-HT-Prot* ≤ *1* +*)*_*Age*≥*70*_, if Age ≥ 70*.*

### Awareness of CKD

Awareness for CKD was calculated as the proportion of participants with eGFR < 60 or CKDu according to the response in the questionnaire if they were aware that they had CKD.

### Statistical analysis

Continuous data are presented as means ± SD. Categorical variables are presented as percent. The CKD prevalence (95% CI) estimates were probability-weighted for the registered 2014 Thai population stratified by age, sex, area of residence (urban/rural), and geographic region. Estimates of means and proportions were calculated for subgroups defined by characteristics of interest. We analyzed the association between risk factors and eGFR < 60 or CKDu with multivariable logistic regression models using *No CKD* as the comparator for all subjects, and CKDu2 and *eGFR* < *60 (DM-HT-Prot* ≤ *1* +*)*_*Age*≥*70*_, separately. Independent variables included age (per 10 years), sex, diabetes (yes/no), hypertension (yes/no), history of gout (yes/no), history of urinary stone (yes/no), taking pain killer (regular/occasional or none), urban/rural, BMI (< 18.5, 18.5– < 25 as reference,25– < 30, and ≥ 30 kg/m^3^), current smoking (yes/no), history of cardiovascular disease (CVD yes/no), and herbal medication use (yes/no). Missing data were excluded. All statistical analyses were performed using Stata version 13.0 software (StataCorp, College Station, TX, USA). The significance level was two-sided and *P* values were set at < 0.05.

## Results

### Characteristics

Of 18,118 participants, 17,329 adults with complete serum creatinine and urinalysis data were included (Figure S1). Mean age was 47 years. 52% were women, 55% were from rural areas, and 56% were farmers/laborers. (Table 1) Diabetes was present in 9.8% and hypertension in 28.5%. In the whole population, eGFR < 60 were older, more likely to live in the rural area with a higher prevalence of women, farmers/laborers, diabetes, hypertension, gout, stones, underweight, herbal medications, painkiller use, and cardiovascular diseases, and lower percentages of secondary education, and smokers compared to *No CKD.* The results were similar when we compared CKDu1 or CKD2 to No CKD in subjects age < 70. (Table 1).

**Table 1:** Characteristics by kidney disease status (all age and under 70).

Parameters	All Ages					Age < 70		
	Total (N = 17,329)	No CKD (N = 15,250)	eGFR < 60 (N = 1415)	eGFR < 60 (DM-HT-) (N = 360)	eGFR < 60 (DM-HT-Prot ≤ 1 +) (N = 351)	No CKD (N = 13,325)	CKDu1 (N = 140)	CKDu2 (N = 136)
Age (years)	47.1 ± 14.6	46.0 ± 14.6	70.3 ± 14.5^a^	68.5 ± 15.9^a^	68.6 ± 15.8^a^	43.9 ± 12.8	57.6 ± 12.8^a^	57.6 ± 12.9^a^
Women (%)	52.0	51.5	63.3^a^	64.6^a^	64.7^a^	51.4	63.3^a^	62.8^a^
Secondary education or higher (%)	42.1	43.7	11.7^a^	16.4^a^	16.5^a^	46.1	25.6^a^	26.0^a^
Rural area (%)	55.1	55.3	61.5^a^	68.3^a^	69.3^a^	55.0	69.1^a^	70.6^a^
Farmer/laborer (%)	55.6	55.8	60.3^a^	70.8^a^	70.4^a^	55.8	77.0^a^	76.8^a^
Diabetes (%)	9.8	8.5	22.9^a^	na	na	8.1	na	na
Hypertension (%)	28.5	26.1	65.0^a^	na	na	24.1	na	na
Gout (%)	2.4	1.7	14.6^a^	12.6^a^	12.4^a^	1.5	17.4^a^	17.2^a^
Stones (%)	3.1	2.9	6.0^a^	8.4^a^	8.5^a^	2.8	13.5^a^	14.0^a^
CVD (%)	3.1	2.8	9.5^a^	6.3^a^	6.4^a^	2.4	6.3^a^	6.5^a^
Painkillers use (%)	2.3	2.2	5.5^a^	9.0^a^	9.2	2.2	12.0^a^	12.4^a^
Herbal medicine %)	25.0	24.6	31.7^a^	28.0^a^	27.5	23.9	28.5^a^	27.2^a^
Current smoker (%)	17.0	21.0	10.0^a^	15.1^a^	15.3^a^	21.4	17.3^a^	17.9^a^
BMI (kg/m^2^)	24.4 ± 4.6	24.4 ± 4.5	23.9 ± 5.6	23.1 ± 4.9^a^	23.1 ± 4.9	24.6 (4.5)	24.1 (4.8)	24.1 (4.9)
**BMI category (%)**								
< 18.5	6.8	6.6	11.0^a^	12.2^a^	12.4^a^	6.0	8.0^a^	8.4^a^
18.5–24.9	53.2	53.4	51.9	60.1	60.2	53.2	56.7	56.9
25–29.9	28.8	28.8	26.6	20.1	19.6	29.2	23.0	22.0
≥ 30	11.3	11.1	10.6	7.65	7.8	11.7	12.3	12.7
Creatinine	0.82 ± 0.36	0.78 ± 0.18	1.5 ± 1.43^a^	1.33 ± 0.86^a^	1.31 ± 0.68^a^	0.78 ± 0.19	1.35 ± 0.50^a^	1.34 ± 0.49^a^
eGFR (ml/min/1.73 m^2^)	99 ± 22	102 ± 18	47 ± 16^a^	50 ± 12^a^	50. ± 12^a^	103. ± 18	51. ± 11^a^	52 ± 10^a^

Mean ± SD; eGFR < 60, decreased GFR-all causes; eGFR < 60 (DM-HT-), decreased GFR without diabetes or hypertension; eGFR < 60 (DM-HT-Prot ≤ 1 +), decreased GFR without diabetes, hypertension or heavy proteinuria; CVD, cardiovascular disease.

^a^*P* < 0.05 versus no CKD.

### The prevalence of CKD and CKDu

**Figure 1:**
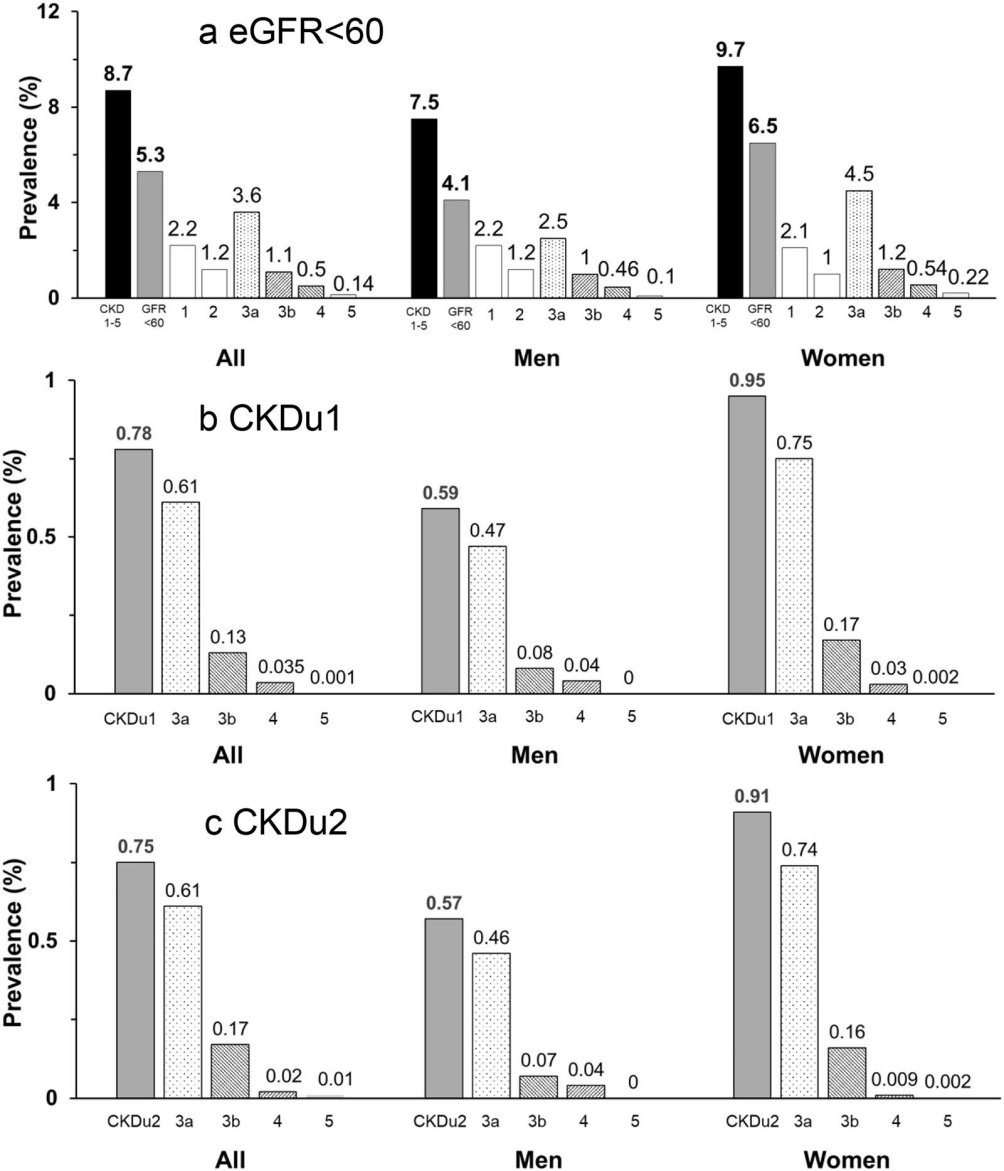
Adjusted Prevalence and Stage of (**a**) CKD1-5, eGFR < 60; (**b**) CKDu1; (**c**) CKDu2 in all adults. Prevalence probability weighted for the registered 2014 Thai population stratified by age, sex, area of residence (urban/rural), and geographic region.

Overall, proteinuria was present in 4.0% (A2, 2.7; A3, 1.3%). Serum creatinine was 0.82 ± 0.36 mg/dl and GFR was 99 ± 22. In men, proteinuria was 3.9% (A2, 2.7; A3 1.2%), serum creatinine was 0.92 ± 0.33 mg/dl, and GFR was 98 ± 19. In women, proteinuria was 4.1% (A2, 2.6; A3 1.4%), serum creatinine was 0.72 ± 0.36. and GFR was 99 ± 25. The GFR distribution by age and sex categories are shown in Figure S2. The stage distribution is shown in Fig. 1 and the population-weighted prevalence (95%CI) of CKD were:

- All, CKD 1–5, 8.7 (8.1–9.3)%; eGFR < 60, 5.3 (4.8–5.8)%; CKDu1, 0.8 (0.7–0.9))%; CKDu2, 0.8 (0.7–0.9)%.
- Men, CKD1–5, 7.5 (6.8–8.3)%: eGFR < 60, 4.1(3.6–4.6)%; CKDu1, 0.6 (0.5–0.8)%; CKDu2 0.6 (0.5–0.7)%.
- Women, CKD1–5, 9.7 (8.6–10.9)%; eGFR < 60, 6.5 (5.4–7.7)%; CKDu1, 1.0 (0.7–1.3)%; CKDu2, 0.9 (0.7–1.2)%.

### Effects of age on CKD prevalence

Table 2 shows GFR and prevalence of CKD by age and sex categories. Overall, 14,437 (83.3%) were age < 70, whereas 2892 (16.7%) were age ≥ of 70. GFR declined with age. eGFR < 60 and Decreased GFR without traditional causes increased with age especially in Age ≥ 70. Women had higher rates for eGFR < 60 and Decreased GFR without traditional causes in nearly all age groups.

**Table 2:** GFR and adjusted prevalence of decreased GFR and CKDu by age and sex.

Age groups	N	eGFR ± SD	eGFR < 60% (N)	eGFR < 60 (DM-HTN-) % (N)	eGFR < 60 (DM-HTN-Prot ≤ 1 +) % (N)
**All subjects**	17,329	99 ± 22	5.3 (1415)	1.5 (360)	1.5 (351)
20–39	3126	115 ± 13	0.20 (8)	0.19 (7)	0.18 (6)
40–49	3333	102 ± 15	0.81 (23)	0.30 (8)	0.30 (8)
50–59	3758	94 ± 15	2.6 (84)	0.70 (21)	0.66 (19)
60–69	4220	82 ± 25	12.2 (437)	3.4 (104)	3.4 (103)
≥ 70	2892	68 ± 26	32.7 (863)	8.8 (220)	8.7 (215)
< 70	14,437	102 ± 20	2.6 (552)	0.78 (140)*	0.75 (136)**
**All men**	7291	98 ± 19	4.1 (573)	1.1 (148)	1.09 (143)
20–39	1284	113 ± 9	0.08 (3)	0.05 (2)	0.05 (2)
40–49	1321	101 ± 12	0.67 (9)	0.23 (3)	0.22 (3)
50–59	1527	93 ± 13	3.4 (42)	0.98 (9)	0.94 (8)
60–69	1853	82 ± 23	8.8 (175)	2.0 (41)	2.0 (41)
≥ 70	1306	69 ± 25	25.1 (344)	7.0 (93)	6.8 (89)
< 70	5985	101 ± 17	2.2 (229)	0.59 (55)*	0.57 (54)**
**All women**	10,038	99 ± 25	6.5 (842)	1.9 (212)	1.8 (208)
20–39	1842	116 ± 15	0.32 (5)	0.32 (5)	0.30 (4)
40–49	2012	102 ± 18	0.94 (14)	0.37 (5)	0.37 (5)
50–59	2231	94 ± 17	1.9 (42)	0.44 (12)	0.39 (11)
60–69	2367	82 ± 27	15.3 (262)	4.7 63)	4.6 (62)
≥ 70	1586	67 ± 26	38.4 (519)	10.1 (127)	10.1 (126)
< 70	8452	103 ± 23	2.9 (323)	0.95 (85)*	0.91(82)**

N = number within group; % = adjusted percent-probability weighted for the registered 2014 Thai population stratified by age, sex, area of residence (urban/rural), geographic region; eGFR < 60, decreased GFR-all causes; eGFR < 60 (DM-HT-), decreased GFR without diabetes or hypertension; eGFR < 60(DM-HT-Prot ≤ 1 +), decreased GFR without diabetes, hypertension or heavy proteinuria.

*CKDu1; ***CKDu2.

### Evaluation using T-GFR and MDRD and comparisons with previous studies

The prevalence of eGFR < 60 and CKDu were higher when MDRD was used and lower when T-GFR was used. (Supplementary Table S1) The higher prevalence rates in women for eGFR < 60 and CKDu were more marked when T-GFR or MDRD were used compared to CKD-EPI.

The prevalence of eGFR < 60 in this study was slightly lower, but within the 95% confidence interval limits of previous recent studies in the Thai general population, (Supplementary Table S2).

### Estimating numbers with CKD

Extrapolating our data to the Thai general population^18^, the estimated numbers with kidney diseases in Thailand are shown in Supplementary Table S3.

### CKD prevalence by region and area

**Figure 2:**
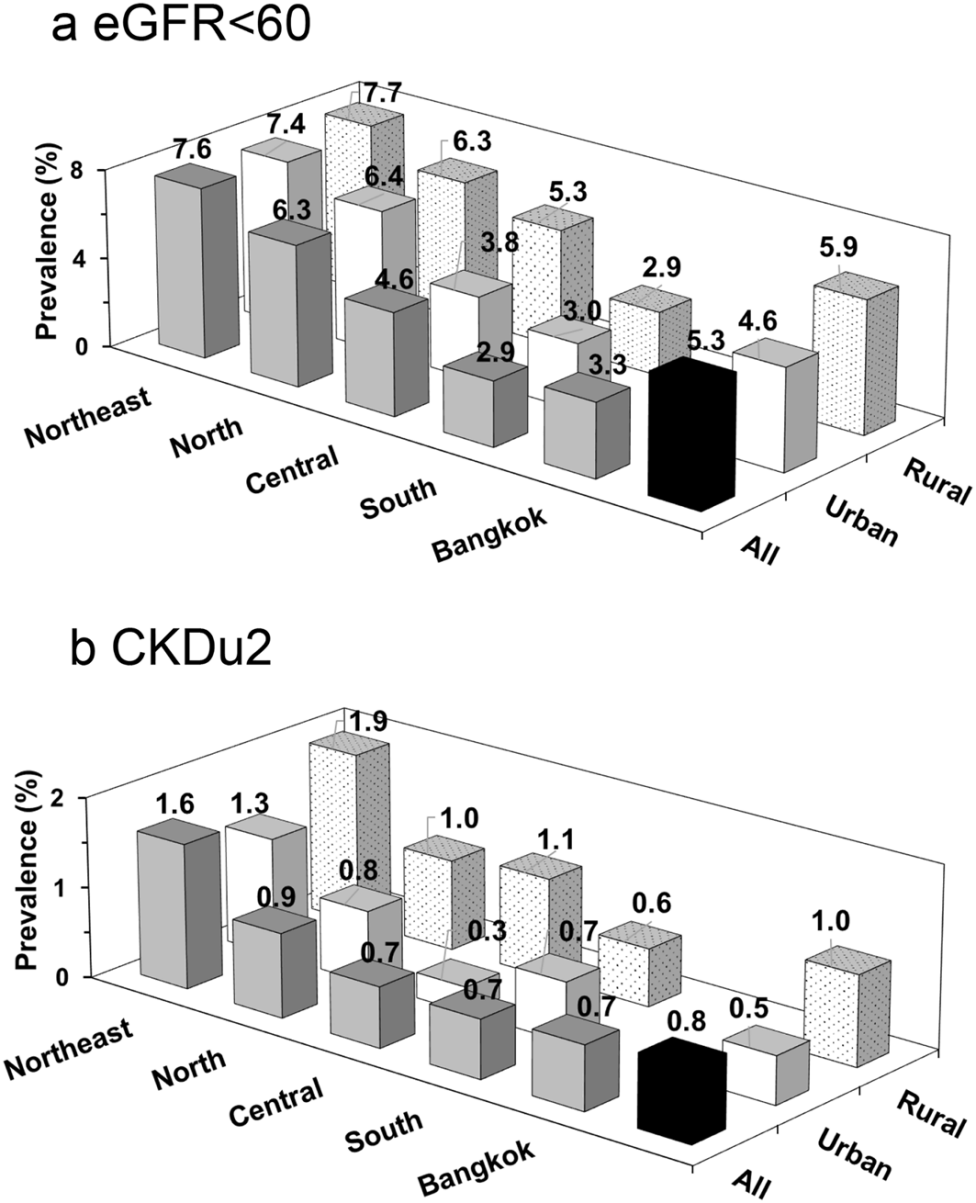
Regional and area distribution of (**a**) eGFR < 60; (**b**) CKDu2. Black column represents total population prevalence. Dark grey column represents combined areas (rural and urban) prevalence for each geographic region. (**a**) eGFR < 60 prevalence shown for all adults (Age ≥ 20): Northeast (n = 3704), North (n = 3896), Central (n = 4240), South (n = 3195), Bangkok (n = 2294). Light column represents urban area (n = 9090) and shaded column represents rural area (n = 8239). (**b**) CKDu2 prevalence shown for adults Age < 70: Northeast (n = 3059), North (n = 3202), Central (n = 3547), South (n = 2602), Bangkok (n = 2025). Light column represents urban area (n = 7684) and shaded column represents rural area (n = 6753).CKDu1 *(not shown)* has nearly identical distribution to CKDu2. Prevalence probability weighted for the registered 2014 Thai population stratified by age, sex, area of residence (urban/rural), and geographic region.

Supplementary Figure S3 shows the different regions (Northeast, North, Central, South, and Bangkok). The prevalence of eGFR < 60 in different regions and areas (rural/urban) are shown in Fig. 2. eGFR < 60 was highest in the Northeast (NE) and lowest in the South and Bangkok with a ratio of 2.5. eGFR < 60 was slightly higher in rural compared to urban areas with an overall ratio of 1.3.

Regional distribution of CKDu generally followed the same pattern as eGFR < 60, but regional differences were more marked with a Northeast: South ratio of 4.3 (Fig. 2 and Supplementary Table S4). The prevalence of CKDu was higher in the rural areas in all regions except the South. Overall, the rural: urban ratio was 1.9–2.0.

#### Effects of age on regional and area distribution

The regional distribution patterns for CKDu and elderly with Decreased GFR without traditional causes were generally similar with the Northeast rural region having the highest rates (Supplementary Table S4). Overall, the rural: urban ratio was greater in CKDu compared to elderly subjects with Decreased GFR without traditional causes.

#### The relative prevalence of CKDu versus traditional causes in age < 70

Supplementary Table S5 shows the ratio of CKDu to eGFR < 60 due to traditional causes (DM, HT, severe proteinuria) in those age < 70. The ratio of CKDu to traditional causes was 0.4 overall, and was higher in the rural compared to the urban areas. The Northeast rural area had among the highest ratio at 0.62.

### Risk factors associated with CKD and CKDu

The frequency of risk factors in eGFR < 60 and CKDu compared to No CKD are shown in Table 1.

#### Multivariable analysis

Education correlated with laborer/farmers occupation and was not included in the model. By multivariable analysis, (Table 3) eGFR < 60 was associated with women, older age, diabetes, hypertension, gout, stone, and painkillers.

**Table 3:** Multivariable analysis of risk factors for decreased GFR and CKDU.

Risk factors	All age	Age < 70		
	eGFR < 60	eGFR < 60	CKDu1	CKDu2
	Odds ratio (95% CI)	Odds ratio (95% CI)	Odds ratio (95% CI)	Odds ratio (95% CI)
Age (per 10 year)	**3.17 (3.14–3.21)**	3.52 (3.42–3.62)	**3.35 (3.42–3.50)**	**3.39 (3.23–3.55)**
Women (men = reference)	**1.70 (1.24–2.34)**	1.65 (1.17–2.32)	**2.41 (1.49–3.89)**	**2.41 (1.49–3.89)**
Diabetes (Y versus N)	**2.01 (1.68–2.40)**	1.67 (1.32–2.10)	na	na
Hypertension (Y versus N)	**2.05 (1.76–2.38)**	2.34 (2.01–2.72)	na	na
Gout (Yes versus No)	**5.69 (4.63–7.00)**	5.58 (4.43–7.02)	**3.77 (2.43–5.84)**	**4.06 (2.65–6.23)**
Kidney stones (Y versus N)	**2.09 (1.52–2.89)**	2.86 (2.18–3.75)	**6.33 (4.24–9.46)**	**6.72 (4.43–10.19)**
Painkillers (regular versus less)	**2.02 (1.51–2.72)**	1.84 (1.26–2.69)	**2.67 (1.16–6.13)**	**2.86 (1.23–6.66)**
Rural (urban = reference)	1.07 (0.92–1.241)	1.16 (0.98–1.37)	**1.66 (1.19–2.32)**	**1.88 (1.32–2.68)**
Laborer/agriculture (other occupations = reference)	1.06 (0.89–1.27)	0.93 (0.77–1.12)	**1.54 (1.08–2.20)**	**1.45 (1.02–2.05)**
**BMI (18.5–24.9 = reference)**				
< 18.5	1.27 (0.91–1.78)	1.56 (1.04–2.36)	**1.86 (1.07–3.21)**	**1.94 (1.12–3.38)**
25–29.9	0.87 (0.75–1.01)	0.79 (0.65–0.97)	0.95 (0.65–1.39)	0.96 (0.64–1.44)
≥ 30	1.03 (0.80–1.33)	0.95 (0.72–1.26)	0.65 (0.33–1.28)	0.69 (0.35–1.37)
Current smoking (non-smoker = reference)	1.14 (0.84–1.55)	1.14 (0.82–1.58)	1.34 (0.85–2.14)	1.36 (0.85–2.18
Cardiovascular disease (Y versus N)	1.19 (0.88–1.61)	1.26 (0.90–1.77)	1.11 (0.44–2.76)	1.16 (0.46–2.95)
Herbal medications (Y versus N)	0.89 (0.79–1.01)	0.89 (0.76–1.04)	0.79 (0.56–1.12)	0.68 (0.46–1.01)

Bold represent statistical significance; Risk compared to no CKD.

Similar to eGFR < 60, women, older age, gout, painkillers were independent risk factors for CKDu. In addition, rural area, stones, low BMI, and laborers/farmers were also significant risk factors for CKDu.

#### Effects of age on risk factors for decreased kidney function without traditional causes

**Figure 3:**
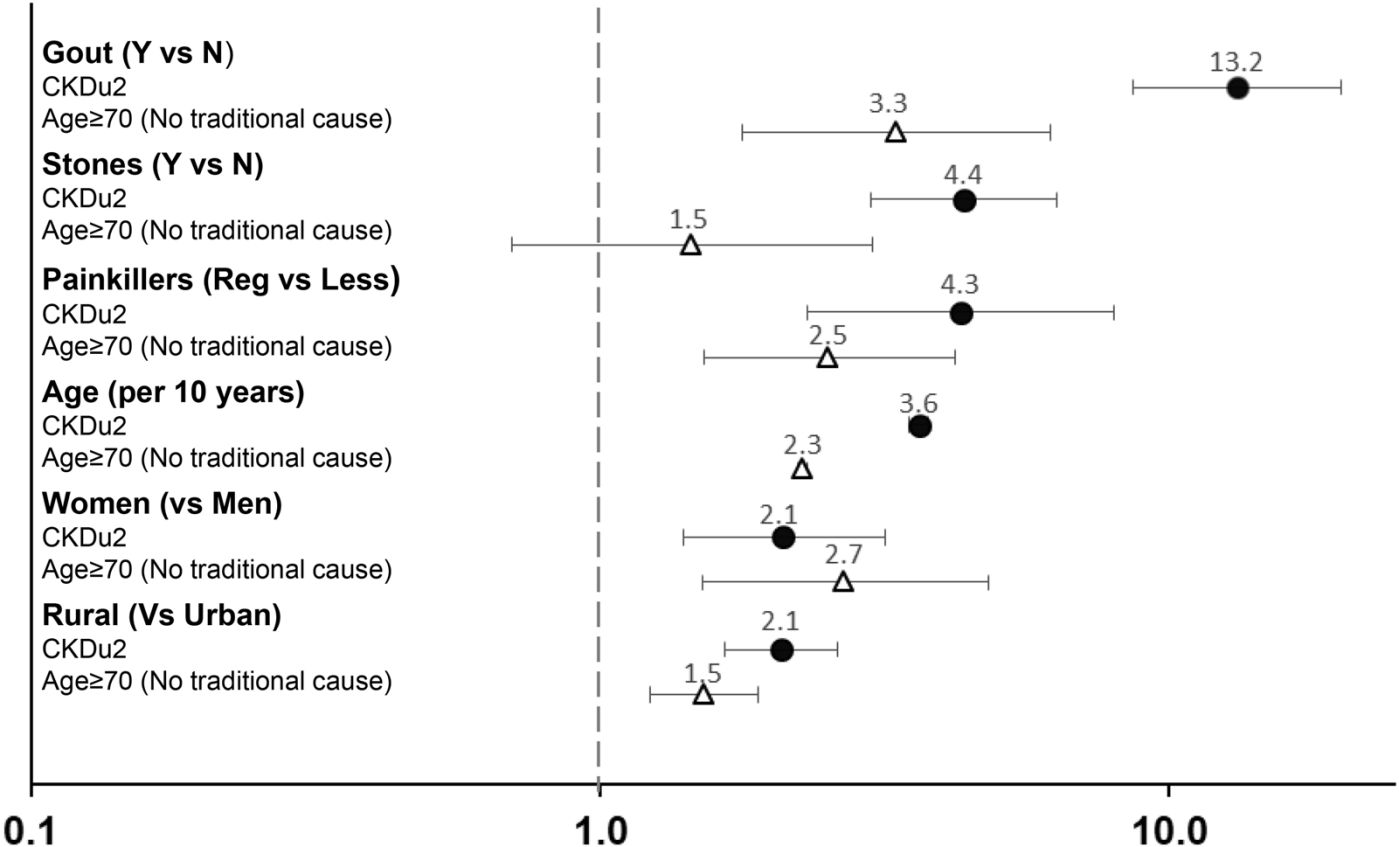
Odds ratio and 95% confidence interval for significant multivariable adjusted risk factors for CKDu2 and elderly subjects with decreased GFR without traditional causes. Odds ratios were calculated separately for CKDu2 (black circle) and subjects age ≥ 70 with decreased GFR without diabetes, hypertension or heavy proteinuria (white triangle). 95% Confidence interval shown as horizontal bars.

To compare risk factors in different age groups, we performed multivariable analysis using the same variables except for occupation as this question was often not answered by the Age ≥ 70. Figure 3 shows odds ratios for risk factors for CKDu2 and *eGFR* < *60 (DM-HT-Prot* ≤ *1* +*)*_*(Age*≥*70)*_. Women, age, rural area, gout, painkillers were significant risk factors for both groups. The odds ratio for gout was higher in CKDu2. Stones were strongly associated with CKDu2, but not for *eGFR* < *60*_*(*_*DM-HT-Prot* ≤ *1* +) _*Age*≥*70)*_*.*

### CKD awareness

CKD awareness was 10.0% (Age < 70, 12.6%; ≥ 70, 7.9%) for eGFR < 60; 5.3% for CKDu1, and 5.5%; for CKDu2.

## Discussion

In this nationally-representative study of the adult Thai population, we found that the prevalence of CKD 1–5, eGFR < 60, CKDu1 and CKDu2 were 8.7%, 5.3%, 0.78%, and 0.75%, respectively. There were considerable regional and area variations, which were more pronounced for CKDu. Hypertension, and/or diabetes were present in over two-thirds with decreased GFR. Excluding these subjects, risk factors for CKDu included women, stones, gout, painkillers, rural area, underweight, and farmer/laborers. The geographic distribution and risk factors for elderly subjects with decreased GFR without traditional causes were generally similar to CKDu. Awareness of CKDu was very low.

Earlier studies found a prevalence of Decreased GFR ranging from 8.6 to 13.6% in the Thai population^9,10,19^. The prevalence of Decreased GFR reported in this study were similar to high income countries^2^. Differences from previous estimates may be due to the differences in the eGFR estimating equation used, creatinine methods, and the period of study^9,10,19^. Our survey was considerably larger and employed IDMS-calibrated enzymatic creatinine method whereas previous studies used the less accurate Jaffe-based method^9,10,19^. All the previous studies employed the MDRD equation as the main eGFR equation. When we recalculated our results using the MDRD equation, the prevalence of eGFR<60 was slightly lower, but within the 95% confidence interval limits of the more recent previous estimates^9^. The high prevalence of Decreased GFR in the Northeast and the low prevalence in the South were consistent with previous surveys^9,10,19^.

The CKDu prevalence in Thailand was considerably lower than older reports from hot spot areas^20^. More recent studies using similar proxy CKDu criteria to our study, showed that the CKDu prevalence in rural communities from Sri-Lanka, India, and Mexico were about 3 to 6 times higher than the Thai population^21–23^. Along with the low absolute numbers, the finding that the ratio of CKDu to decreased GFR due to known causes was less than 1 provides further support that CKDu was less of a problem in Thailand compared to endemic regions. By comparison, this ratio was above 1 in Sri Lanka^21^, and the prevalence of eGFR < 60 may reach 18% among subjects under age 60 in some Central American communities even when diabetes and hypertension were uncommon^24^. The CKDu prevalence in Thailand showed 2–4 folds regional and area variations with urban areas being comparable to the US population-based estimates of eGFR < 60 without hypertension or diabetes^25^. Recent improvement in living conditions and healthcare services may have reduced CKDu overall, but socioeconomic inequalities and differences in exposure to risk factors may account for the differences between urban and rural areas and between regions. Higher CKDu prevalence in the Northeast, especially in the rural area may reflect hotter climate, higher rates of poverty, high frequency of stones, and greater reliance on farming, and manual labor in this region^26^.

Consistent with current hypotheses, Thai farmers/laborers and those living in the rural area had increased CKDu^3,7,20^. The equatorial climate predisposes farmers and laborers to dehydration, which may be an important mechanism for CKDu^3^. Other factors such as pesticides and heavy metals may contribute^3^. Herbicides such as paraquat and glyphosate, were widely used in Thailand^27^. Thai farmers may have greatly elevated urinary pesticide levels^28^, and pesticide exposure has been associated with increased tubular injury^29^. Nonetheless, the role of pesticides as a cause for CKDu remains uncertain^27^. Heavy metals such as lead, cadmium, and arsenic are well-established nephrotoxins^3,30^. Thai villagers living in areas contaminated by waste from zinc mines had markedly elevated urinary cadmium and decreased GFR^31^. In other areas, the groundwater or shallow well water, which were used for consumption in parts of rural Thailand may become contaminated by lower levels of heavy metals from chemical fertilizers^32^. However, industrial contamination is restricted to certain areas^25^, and the role of lower environmental exposure of heavy metals as causes of CKDu remains unclear^30^.

Gout, kidney stones, and painkillers were strongly associated with both Decreased GFR and CKDu. The nephrotoxicity of these factors may be enhanced by dehydration. Hyperuricemia has been associated with CKD and CKDu in several populations^33,34^. Elevated serum uric acid and uric acid crystalluria may promote kidney injury^35^. Allopurinol may reduce heat stress-induced renal injury in experimental models^36^, but the clinical benefit remains to be demonstrated in CKDu. Research studies have demonstrated a role for nephrolithiasis in the pathogenesis of CKD^37^. Recurrent obstruction, infections, and structural abnormalities may be potential mechanisms. NSAID use has been associated with CKDu in some Central American communities but was not a primary etiologic factor^20^. Our survey did not differentiate NSAID from other types of painkillers, but the impact of regular painkillers (and presumably NSAID) use on CKDu was more marked in the Thai population. Low body mass was a borderline risk factor for CKDu. Underweight subjects had been shown to have increased the risk for kidney failure in China^38^. Low body mass may reflect intercurrent illness or maybe a surrogate marker for the most socially deprived^10^.

Previous data from Thailand^9,10^ and other countries^2^, have consistently shown higher risk of Decreased GFR in women. On the other hand, men have been shown to be at greater risk for CKDu in Central America or South Asia^3,7,20^. In these regions, men performed most of the agricultural and labor work. Unlike many areas around the world, Thai women often share the strenuous work in farming and manual labor^39^. With similar exposure to environmental risk factors, the higher CKDu prevalence may reflect as yet unknown risk factors in Thai women. Part of the higher rates of CKDu in Thai women may reflect the fact that the CKD-EPI formulae despite sex being included as a variable in the equation may underestimate the true GFR in women more often than in men^40^. Nonetheless, it is unlikely that bias in the CKD-EPI equation alone could fully account for the higher prevalence in women as the sex differences were even more marked when MDRD or T-GFR equations were used to calculate eGFR. Similar to previous studies, decreased GFR without traditional causes were uncommon before the age of 50^21^, and increased exponentially over age 70. Both prolonged exposure to the same risk factors that cause CKDu, and aging-related nephron loss likely contributed to the high prevalence of reduced GFR in the elderly^41^.

This study has several strengths and implications. This was the largest representative survey of CKD prevalence in Thailand, and to our knowledge, the only population-based study to evaluate the prevalence and risk factors for CKDu in Southeast Asia. The study employed a standardized protocol and optimized creatinine measurement method as proposed by i3C workgroup. The multistep sampling study design should enable the results to be extrapolated to the Thai general population. The regional variations of CKDu in Thailand may serve as a model for other LMIC countries where overall socioeconomic development has been considerable but unevenly distributed. Our data enables the identification of local hot spots so that healthcare resources can be allocated to subpopulations at risk^26^. The finding that about one-third of patients with decreased GFR fulfilled the proxy criteria for CKDu has implications for prevention. Past screening strategies, which focused mainly on hypertensive and diabetic subjects, may miss CKDu. This is especially important since the awareness for CKDu was less than 5%. Risk factors such as painkillers, renal stones, or gout may be amenable for intervention or prevention. This study should stimulate research into the causes of CKDu in Thailand and why women have elevated risks. Finally, given the similarity in risk factors and regional distribution of CKDu and decreased GFR without traditional causes in the elderly, the optimal age cut-off to classify CKDu remains to be defined. It is probable that cumulative exposure to risk factors that cause CKDu may enhance the age-related GFR decline.

There are several limitations. Firstly, we used proxy definitions based on one-time measurement of kidney function. By not confirming the presence of abnormal kidney function for greater than 3 months, this study likely overestimates true CKD since some subjects would have reversible abnormalities. The i3C workgroup accepts this limitation as these definitions allow a reasonable estimate of CKDu in population-level surveys given the difficulty in repeating the kidney function in large epidemiological studies^6^. Second, we used CKD-EPI to calculate eGFR as this is the preferred equation for general population studies^14,17^, and is the equation recommended by the Nephrology Society of Thailand^6^. Nonetheless, CKD-EPI has not been validated formally in the Thai general population. We also calculated our results using T-GFR^16^. T-GFR was developed in Thai CKD subjects, and its accuracy in the general population is unclear. For the reference GFR method, T-GFR used plasma clearance of Tc-99 m DTPA (diethylene-triamine-pentaacetate), which is known to overestimate GFR compared to inulin clearance^42^. This bias could account for the lower prevalence of eGFR < 60 with T-GFR equation as has been reported in previous studies^13^. In the absence of an ideal equations for the Thai general population, we followed the recommendations of ISN i3C group and used CKD-EPI as our main equation, but acknowledge the existence of potential bias that may affect our results. Thirdly, we used dipstick protein rather than urine protein creatinine ratio, which would be too costly. Previous studies have shown fairly close correlations of dipstick protein with quantitative measurements^17^. Finally, this survey did not contain detailed questions on water supplies and toxin exposure which might have increased our understanding on CKDu risk factors.

In conclusion, although the prevalence of CKDu in Thailand was low overall, there remained areas with higher risk. Our study may be a model for higher-middle income country, where despite overall economic and healthcare improvement, much work needs to be done to increase the awareness and reduce the risk for CKDu in underserved regions. Although there were some differences, risk factors for CKDu were generally similar to older subjects with decreased GFR without traditional causes. Unlike other countries, Thai women had an increased risk of CKDu. Future studies are necessary to identify causes of CKDu in high-risk groups in Thailand.

## Supplementary Information

Supplementary Information.
