# Author Correction: Women and other risk factors for chronic kidney disease of unknown etiology in Thailand: National Health Examination V Survey

**DOI:** 10.1038/s41598-021-02191-5

**Published:** 2021-11-16

**Authors:** Wichai Aekplakorn, Suwat Chariyalertsak, Pattapong Kessomboon, Sawitri Assanangkornchai, Surasak Taneepanichskul, Nareemarn Neelapaichit, Anchalee Chittamma, Chagriya Kitiyakara

**Affiliations:** 1 Department of Community Medicine, Faculty of Medicine, Ramathibodi Hospital, Mahidol University, Bangkok, Thailand; 2 Faculty of Public Health, Chiang Mai University, Chiang Mai, Thailand; 3 Department of Community Medicine, Faculty of Medicine, Khon Kaen University, Khon Kaen, Thailand; 4 Epidemiology Unit, Faculty of Medicine, Prince of Songkla University, Songkhla, Thailand; 5 College of Public Health Sciences, Chulalongkorn University, Bangkok, Thailand; 6 Ramathibodi School of Nursing, Faculty of Medicine, Ramathibodi Hospital, Mahidol University, Bangkok, Thailand; 7 Department of Pathology, Faculty of Medicine, Ramathibodi Hospital, Mahidol University, Bangkok, Thailand; 8 Department of Medicine, Faculty of Medicine, Ramathibodi Hospital, Mahidol University, Bangkok, Thailand

Correction to: *Scientific Reports* 10.1038/s41598-021-00694-9, published online 01 November 2021

The original version of this Article contained an error in Affiliation 6, which was incorrectly given as ‘Faculty of Medicine, Ramathibodi School of Nursing, Ramathibodi Hospital, Mahidol University, Bangkok, Thailand’. The correct affiliation is listed below:

Ramathibodi School of Nursing, Faculty of Medicine, Ramathibodi Hospital, Mahidol University, Bangkok, Thailand

The original Article has been corrected.

