# Supplementary material for: Women and other risk factors for chronic kidney disease of unknown etiology in Thailand: National Health Examination V Survey
Source: Sci Rep. 2021 Nov 1;11:21366. doi: 10.1038/s41598-021-00694-9 (PMC8560950; doi:10.1038/s41598-021-00694-9)
Supplement: Supplementary file 1 — Supplementary Information. [file 41598_2021_694_MOESM1_ESM.pdf]

Supplementary Materials

Women and other risk factors for chronic kidney disease of unknown etiology in Thailand: National Health Examination V Survey

Wichai Aekplakorn MD, PhD[1]; Suwat Chariyalertsak, MD, DrPH[2]; Pattapong Kessomboon, MD, PhD[3]; Sawitri Assanangkornchai MD, PhD[4]; Surasak Taneepanichskul, MD, MMed[5]; Nareemarn Neelapaichit, PhD[6]; Anchalee Chittamma PhD[7], Chagriya Kitiyakara MBBS FRCP[8*]

1.Department of Community Medicine, Faculty of Medicine, Ramathibodi Hospital, Mahidol University, Bangkok, Thailand
2 Faculty of Public Health, Chiang Mai University, Chiang Mai, Thailand
3. Department of Community Medicine, Faculty of Medicine, Khon Kaen University, Khon Kaen, Thailand
4. Epidemiology Unit, Faculty of Medicine, Prince of Songkla University, Songkhla, Thailand
5. College of Public Health Sciences, Chulalongkorn University, Bangkok, Thailand
6. Ramathibodi School of Nursing, Faculty of Medicine, Ramathibodi Hospital, Mahidol University, Bangkok, Thailand
7. Department of Pathology, Faculty of Medicine, Ramathibodi Hospital, Mahidol University Bangkok, Thailand
8. Department of Medicine, Faculty of Medicine, Ramathibodi Hospital, Mahidol University, Bangkok, Thailand

*** Corresponding author:**
Chagriya Kitiyakara MBBS FRCP.
Department of Medicine, Faculty of Medicine, Ramathibodi Hospital, Rama VI Rd., Ratchathewi, Bangkok 10400, Thailand. Tel: 662-2011400, Fax: 662-201-1400. Email: kitiyakc@yahoo.com

Supplementary Tables: 5

Supplementary Figures: 3

Acknowledgement

Supplementary tables

Table S1: Prevalence of CKD and CKDu using different GFR estimating equations

	All	Age<70		Age≥70	
Equation	Men+Women	Men	Women	Men	Women
**CKD-EPI**					
CKD1-5	8.7 (8.1-9.3)	7.5 (6.8-8.3)	9.7 (8.6-10.9)	28.4 (25.5-31.6)	41.1(35.3 -47.2)
eGFR<60	5.3 (4.8-5.8)	4.1(3.6-4.6)	6.5 (5.4-7.7)	25.1 (22.4 -28.0	38.4 (32.4 -44.7)
CKDu1	0.8 (0.7-0.9)	0.6 (0.5 -0.8)	1.0 (0.7-1.3)	na	na
CKDu2	0.8 (0.7-0.9)	0.6 (0.5-0.7)	0.9 (0.7-1.2)	na	na
**MDRD**					
CKD1-5	11.3 (10.0-13.1)	6.3 (5.6, 7.1)	11.2 (8.4-14.8)	27.4 (24.6-30.5)	42.8 (37.2-48.6)
eGFR<60	8.1 (6.5-10.0)	2.9 (2.5-3.3)	8.0 (5.3-11.9)	24.1 (21.5-26.8)	40.2 (34.4-46.3)
CKDu1	2.4 (1.6-3.5)	0.9 (0.7-1.1)	3.8 (2.3-6.1)	na	na
CKDu2	2.3 (1.6-3.5)	0.9 (0.7- 1.1)	3.7 (2.3-6.1)	na	na
**T-GFR**					
CKD1-5	8.3 (7.6-9.1)	4.8 (4.2, 5.4)	6.7 (5.9, 7.7)	19.5 (17.3, 21.9)	44.0 (38.5, 49.6)
eGFR<60	4.9 (4.3-5.6)	1.2 (1.0-1.4)	3.5 (2.8-4.3)	15.5 (13.6-17.5)	41.3 (35.6-47.2)
CKDu1	0.7 (0.6-1.0)	0.2 (0.2-0.4)	1.2 (0.8-1.7)	na	na
CKDu2	0.7 (0.5-0.9)	0.2(0.2-0.4)	1.1 (0.8-1.6)	na	na

Data as % (95% Confidence Interval) - probability weighted for the registered 2014 Thai population stratified by age, sex, area of residence (urban/rural), geographic region

CKD-EPI, Chronic Kidney Disease Epidemiology Collaboration; MDRD, Modification of Diet in Renal Disease; T-GFR= Thai GFR

CKDu=Age<70 only

Table S2: Population-based studies of Decreased GFR in Thailand

Study	Year of survey	N	Age	Creatinine Method, Standardization	eGFR Equation	eGFR<60 (%)	Northeast region highest	First Author (Ref)
InterAsia	2000	5146	>35	Modified Jaffe, Standardized to NHANES serum	MDRD-	13.8	Yes	Perkovic, V (10)
NHES III	2004	3117	>15	Modified Jaffe	MDRD	8.9	Yes	Ong-Ajyooth, L (19)
Thai-SEEK	2007	3459	>18	Modified Jaffe, IDMS	MDRD<br>CKD-EPI	8.6<br>6.5	Yes	Ingsathit, A (9)
NHES V	2014	17,329	>20	Enzymatic, IDMS	MDRD<br>CKD-EPI	8.1<br>5.3	Yes	Present study

IDMS, isotope dilution mass spectrometry traceable standard;

CKD-EPI, Chronic Kidney Disease Epidemiology Collaboration; MDRD, Modification of Diet in Renal Disease

Table S3: Estimated number (95% CI) of adults with kidney diseases in Thailand

	All	Age<70		Age≥70	
Kidney Disease	Men+Women	Men	Women	Men	Women
CKD1-5	3,900,080 (3,383,667-4,416,493)	1,125,126 (974,819-1,275,433)	1,309,245 (1,084,480-1,534,011)	498,977 (426,468-571,486)	966,732 (752,211-1,181,252)
eGFR<60	2,387,062 (2,025,452 -2,748,672)	436,578 (375,925-497,230)	608,151 (477,445-738,857)	440,207 (373,351-507,064)	902,126 (688,330-1,115,921)
CKDu1	321,832 (258,009- 385,654)	117,971 (89,891- 146,051)	203,861 (141,864-65,858)	na	na
CKDu2	310,595 (248,166-373,002)	115,491 (87,272-143,711)	195,104 (133,973-256,234)	na	na

Estimates based on Thai National Census data in 2014 based on 48,152,153 adults (age ≥20) (Men, 22,940,105; Women, 25,212,048) probability weighted for age, sex, area of residence (urban/rural), geographic region
CKDu = Age <70 only

Table S4: Regional or area distribution of CKD by age groups

	Age <70			Age≥70		
	N	CKDu1 (%)	CKDu2 (%)	N	eGFR<60 (DM-HTN-) (%)	eGFR<60 (DM-HTN-Prot≤1+) (%)
Region						
Northeast	3,059	1.3	1.3	645	13.8	13.8
Rural		1.6	1.6	339	15.0	14.9
Urban		0.8	0.8	306	11.2	11.0
North	3,202	0.99	0.75	694	8.0	8.0
Rural		1.0	0.9	388	9.6	9.6
Urban		0.7	0.5	306	4.9	4.9
Central	3,547	0.69	0.5	693	6.7	6.6
Rural		0.8	0.8	370	7.0	7.0
Urban		0.2	0.1	323	6.4	6.1
South	2,602	0.3	0.3	593	4.5	4.3
Rural		0.3	0.3	389	4.9	4.9
Urban		0.4	0.3	204	3.8	3.1
Bangkok	2,025	0.73	0.71	267	7.7	7.7
**NE:S ratio**		**4.3**	**4.3**		**3.1**	**3.2**
Area						
All Rural	6,753	1.0	1.0	1486	10.2	10.1
All Urban	7,682	0.53	0.50	1406	7.2	7.0
**Rural: Urban ratio**		**1.9**	**2**		**1.4**	**1.4**

% = Adjusted Percent- probability weighted for the registered 2014 Thai population stratified by age, sex, area of residence (urban/rural), geographic region
eGFR<60 (DM-HTN-), Decreased GFR without diabetes or hypertension; eGFR<60 (DM-HTN-Prot≤1+), Decreased GFR without diabetes or hypertension or heavy proteinuria

Table S5: Ratio of CKDu to eGFR<60 with traditional factors by region in subjects Age <70

	N	eGFR<60 (DM and/or HT) (%)	CKDu1 (%)	**Ratio** CKDu1 to eGFR<60 (DM and/or HT)	eGFR<60 (DM and/or HTN and/or Protein≥2+) (%)	CKDu2 (%)	**Ratio** CKDu2 to eGFR<60 (DM and/or HTN and/or Protein≥2+)
Region							
Northeast	3,059	2.5	1.3	0.52	2.5	1.3	0.52
Rural		2.6	1.6	0.62	2.6	1.6	0.62
Urban		2.4	0.8	0.33	2.4	0.8	0.33
North	3,202	2.2	0.99	0.45	2.3	0.75	0.33
Rural		2.1	1.0	0.48	2.2	0.9	0.41
Urban		2.3	0.7	0.30	2.5	0.5	0.20
Central	3,547	1.5	0.69	0.46	1.5	0.5	0.33
Rural		1.6	0.8	0.50	1.6	0.8	0.50
Urban		1.5	0.2	0.13	1.5	0.1	0.07
South	2,602	1.0	0.3	0.30	1.0	0.3	0.30
Rural		1.0	0.3	0.30	0.9	0.3	0.33
Urban		1.2	0.4	0.33	1.2	0.3	0.25
Bangkok	2,025	1.0	0.73	0.73	1.0	0.71	0.71
Area							
All Rural	6,753	1.9	1.0	0.53	2.0	1.0	0.50
All Urban	7,684	1.6	0.53	0.33	1.6	0.50	0.31
**ALL**	**14,437**	**1.8**	**0.79**	**0.44**	**1.8**	**0.76**	**0.42**

% = Adjusted Percent- probability weighted for the registered 2014 Thai population stratified by age, sex, area of residence (urban/rural), region
eGFR<60(DM and/or HT), Decreased GFR with hypertension or diabetes mellitus; eGFR<60 (DM and/or HTN and/or Protein≥2+), Decreased GFR with hypertension or diabetes mellitus or heavy proteinuria.

Supplementary Figures

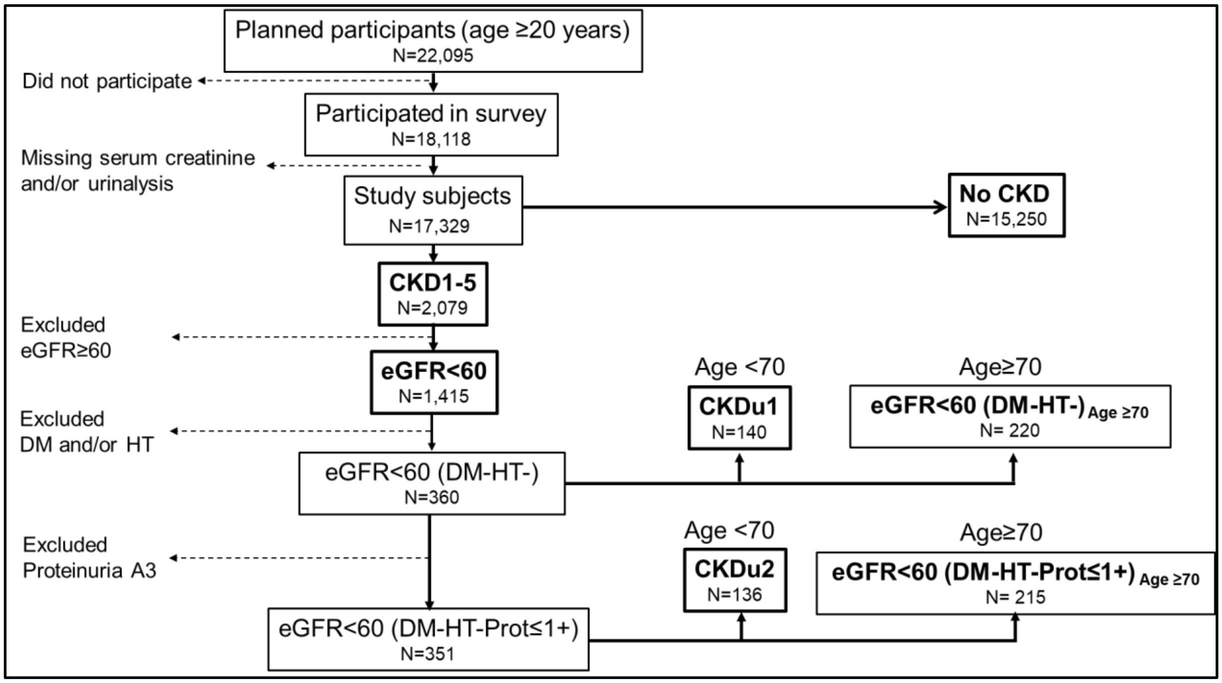

Figure S1: Flow diagram showing subjects with kidney diseases

eGFR<60 (DM-HT-), Decreased GFR without hypertension or diabetes**;** eGFR<60 (DM-HT-Prot≤1), Decreased GFR without hypertension or diabetes or heavy proteinuria.

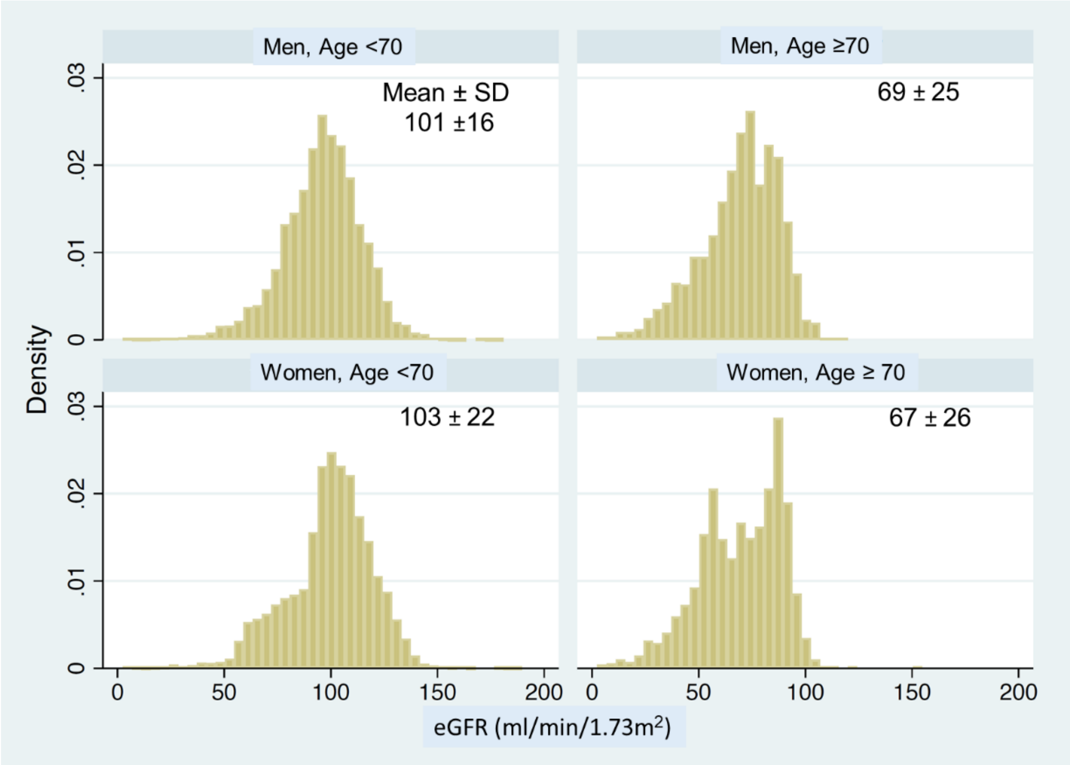

Figure S2: Distribution of adult Thai population eGFR stratified by age and sex.

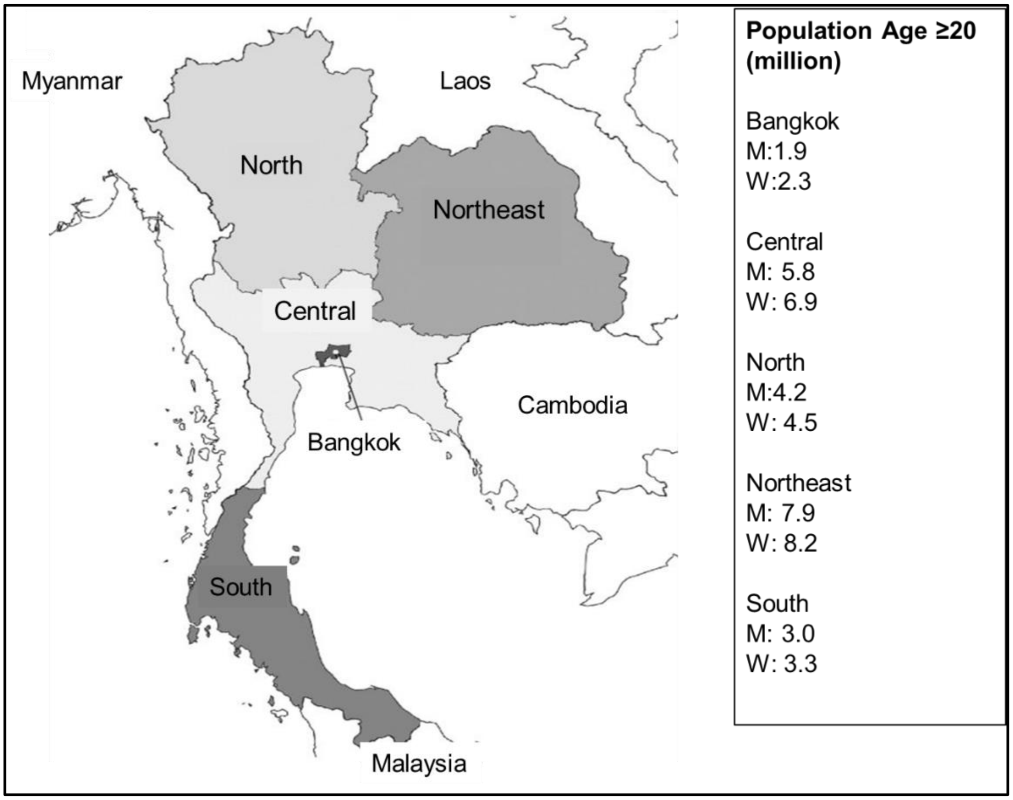

Figure S3: Map of Thailand regions and population distribution

Acknowledgement

NHES study team members, National Health Examination Survey Office, Department of Community Medicine, Faculty of Medicine Ramathibodi Hospital: Wichai Aekplakorn, Jiraluck Nonthaluck, and Nareemarn Neelapaichit; **Northern region**: Suwat Chariyalertsak, Kanittha Thaikla (Chiang Mai University), Sutthinan Srathonghon, Ratana Phanphanit, Jiraporn Suwanteerangkul, and Kriangkai Srithanaviboonchai; **North Eastern Region:** Pattapong Kessomboon, Piyathida Kuhirunyaratn, Sauwanan Bumrerraj, Bangornsri Jindawong, and Weerapong Seeupalat (Khon Kaen University); **Southern region:** Sawitri Assanangkornchai, Virasakdi Chongsuvivatwong, and Darika Saingam (Prince of Songkla University); **Central Region:** Surasak Taneepanichskul**,** Somrat Lertmaharit**,** Vilai Chinveschakitvanich**,** Onuma Zongram**,** Nuchanad Hounnaklang**,** and Sukarin Wimuktayon (Chulalongkorn University); **Bangkok Region:** Panwadee Putwatana, Chalermsri Nuntawan, and Karn Chaladthanyagid (Mahidol University).
